# Integrated physiological and weighted gene co-expression network analysis reveals the hub genes engaged in nitrate-regulated alleviation of ammonium toxicity at the seedling stage in wheat (*Triticum aestivum* L.)

**DOI:** 10.3389/fpls.2022.1012966

**Published:** 2022-11-17

**Authors:** Liuyin Li, Xiuzhi Zang, Jianbo Liu, Jinfeng Ren, Zhenlin Wang, Dongqing Yang

**Affiliations:** College of Agronomy, Shandong Agricultural University/State Key Laboratory of Crop Biology, Tai’an, Shandong, China

**Keywords:** wheat seedlings, transcriptome, weighted gene co-expression network, nitrate, ammonium toxicity

## Abstract

Wheat has a specific preference for NO_3_^-^ and shows toxicity symptoms under high NH_4_^+^ concentrations. Increasing the nitrate supply may alleviate ammonium stress. Nevertheless, the mechanisms underlying the nitrate regulation of wheat root growth to alleviate ammonium toxicity remain unclear. In this study, we integrated physiological and weighted gene co-expression network analysis (WGCNA) to identify the hub genes involved in nitrate alleviation of ammonium toxicity at the wheat seedling stage. Five NH_4_^+^/NO_3_^-^ ratio treatments, including 100/0 (N_a_), 75/25 (N_r1_), 50/50 (N_r2_), 25/75 (N_r3_), and 0/100 (N_n_) were tested in this study. The results showed that sole ammonium treatment (N_a_) increased the lateral root number but reduced root biomass. Increasing the nitrate supply significantly increased the root biomass. Increasing nitrate levels decreased abscisic acid (ABA) content and increased auxin (IAA) content. Furthermore, we identified two modules (blue and turquoise) using transcriptome data that were significantly related to root physiological growth indicators. *TraesCS6A02G178000* and *TraesCS2B02G056300* were identified as hub genes in the two modules which coded for plastidic ATP/ADP-transporter and *WRKY62* transcription factors, respectively. Additionally, network analysis showed that in the blue module, *TraesCS6A02G178000* interacts with downregulated genes that coded for indolin-2-one monooxygenase, SRG1, DETOXIFICATION, and wall-associated receptor kinase. In the turquoise module, *TraesCS2B02G056300* was highly related to the genes that encoded *ERD4*, *ERF109*, *CIGR2*, and WD40 proteins, and transcription factors including *WRKY24*, *WRKY22*, *MYB30*, and *JAMYB*, which were all upregulated by increasing nitrate supply. These studies suggest that increasing the nitrate supply could improve root growth and alleviate ammonium toxicity through physiological and molecular regulation networks, including ROS, hormonal crosstalk, and transcription factors.

## Introduction

Wheat (*Triticum aestivum* L.) is an important cereal crop worldwide. Nitrogen (N) is a crucial limiting nutrient that has a central effect on crop growth and yield formation. NO_3_^-^ and NH_4_^+^ are the most prominent forms of inorganic N taken up by plant roots (Feng et al., 2020). In agricultural fields, the use of nitrification inhibitors together with ammonium fertilizers or organic fertilizers stabilizes ammonium at high concentrations in the soil for several weeks (Marino and Moran, 2019). Nevertheless, wheat has a specific preference for NO_3_^-^ and shows toxicity symptoms under high NH_4_^+^ concentrations, including reduced plant growth, decreased root length, and leaf chlorosis (Imran et al., 2019).

Previous studies have demonstrated a few physiological and molecular mechanisms underlying the toxicity of NH_4_^+^ stress. Firstly, endogenous hormones can be imbalanced by an excess supply of ammonium. A previous study reported that high ammonium concentrations increased ethylene production (Lynch and Brown, 1997). Yang et al. (2015) found that the expression of auxin-regulated genes is repressed in NH_4_^+^ -stressed plants. Furthermore, abscisic acid (ABA) plays a positive role in regulating the *OsSAPK9*–*OsbZIP20* pathway in rice to increase tolerance to high-NH_4_^+^ stress (Sun et al., 2020). Britto et al. (2001) proposed that the transportation of NH_4_^+^ across membranes was energy-intensive when high concentrations of ammonium were present in the medium, resulting in massive ATP consumption and waste. On the other hand, NH_4_^+^ excess causes inhibition cation uptake, such as K^+^ and Mg^2+^, which consequently change plant ion balance (Esteban et al., 2016). A recent study found that excessive assimilation of ammonium by plastidic glutamine synthetase produces high levels of protons and aggravates the acidic burden that leads to plant toxicity (Hachiya et al., 2021). NO_3_^-^ has positive effects on hormone synthesis and transport to recover from the negative effects of ammonium stress (Liu et al., 2019). *Sucrose non-fermenting-1-related protein kinase* (*SnRK1.1*) negatively regulates the nitrate channel *SLOW ANION CHANNEL Homologue 3* (*SLAH3*), which is involved in nitrate-dependent alleviation of ammonium toxicity (Sun et al., 2021). Xiao et al. (2022) revealed that *NRT1.1* and *SLAH3* could form a functional unit to regulate nitrate-dependent alleviation of ammonium toxicity by regulating NO_3_^-^ transport and balancing rhizosphere acidification. In our previous study, we reported that a high NH_4_^+^/NO_3_^-^ ratio enhances the expression of genes and proteins involved in lignin biosynthesis, leading to root lignification, thereby resulting in increased root oxidative tolerance at the cost of reducing nitrate transport and utilization (Yang et al., 2022). These results suggest that increasing nitrate levels may alleviate ammonium toxicity. These studies have succeeded in determining the physiological and molecular components associated with ammonium toxicity. Nevertheless, the molecular mechanisms underlying the nitrate regulation of wheat root growth to alleviate ammonium toxicity remain unclear.

In the post-genomic era, omics and bioinformatics are essential for understanding the complex regulatory networks in plants associated with stress adaptation and tolerance (Urano et al., 2010; Mochida and Shinozaki, 2011). For example, a previous study integrated transcriptome and metabolome analyses to reveal the physiological and molecular responses of rapeseed to ammonium toxicity (Li et al., 2021). Weighted gene co-expression network analysis (WGCNA) is widely used to identify hubs in biological systems, including plant responses to abiotic stresses (Randhawa and Pathania, 2020; Di Silvestre et al., 2021). WGCNA was used to explore candidate hub genes involved in drought adaptation in wheat (Luo et al., 2019). A study by Chen et al. (2021) showed that WGCNA could be performed to identify hub genes, networks, and pathways relevant to whole leaf responses to waterlogging stress. Integrated WGCNA and physiological analysis may provide new insights to uncover hub genes involved in nitrate alleviation of ammonium toxicity. The objectives of the present study were to (i) investigate the wheat root transcriptome profiles in response to different NH_4_^+^/NO_3_^-^ ratios treatments, (ii) identify functional gene module networks that are involved in the root response to ammonium stress by WGCNA analysis, and (iii) provide new insights into nitrate alleviation of ammonium toxicity.

## Materials and methods

### Plant materials and culture conditions

The experiments were conducted at the experimental station of Shandong Agricultural University, Tai’ an, China. The Jimai 22 winter wheat cultivar was grown under controlled conditions. Variations in the temperature, illumination intensity, and relative humidity are shown in **Supplementary Figure S1**. Seeds were surface-sterilized with 1% NaClO_3_ for 30 min, rinsed five× with sterile water, and germinated on wet filter paper in the dark at 25°C for two days. Germinated seeds were sown in plastic pots (10 × 10 × 10 cm; 10 plants/pot) filled with perlite. The pots were placed on plastic trays (55 × 45 × 5 cm; 20 pots/tray).

### Treatments and experimental design

Five NH_4_^+^/NO_3_^-^ ratio treatments with a total N concentration of 6 mM were used to investigate how nitrate alleviated ammonium toxicity in a controlled climate chamber experiment. Treatments included 100/0 (N_a_), 75/25 (N_r1_), 50/50 (N_r2_), 25/75 (N_r3_), and 0/100 (N_n_). Ca(NO_3_)_2_, NH_4_Cl, and NH_4_NO_3_ were used to set these ratios. The NH_4_^+^/NO_3_^-^ ratios were determined according to our previous study (Yang et al., 2022). The NH_4_^+^/NO_3_^-^ ratios preparation according to the following method: 6 mmol L^-1^ NH_4_Cl and 4 mmol L^-1^ CaCl_2_ for N_a_; 4.5 mmol L^-1^NH_4_Cl, 0.75 mmol L^-1^ Ca(NO_3_)_2_ and 3.25 mmol L^-1^ CaCl_2_ for N_r1_; 3 mmol L^-1^ NH_4_NO_3_ and 4 mmol L^-1^ CaCl_2_ for N_r2_; 1.5 mmol L^-1^NH_4_Cl, 2.25 mmol L^-1^ Ca(NO_3_)_2_ and 1.75 mmol L^-1^ CaCl_2_ for N_r3_; 3 mmol L^-1^ Ca(NO_3_)_2_ and 1 mmol L^-1^ CaCl_2_ for N_n_. The concentration of all other nutrient elements was referred to a modified Hoagland’s nutrient solution with the following chemical composition: 5 mmol·L^-1^ KCl, 4 mmol·L^-1^ MgSO_4_, 1 mmol·L^-1^ KH_2_PO_4_, 50 μmol·L^-1^ Fe-EDTA, 0.5 μmol·L^-1^ H_3_BO_3_, 0.74 μmol·L^-1^ MnSO_4_, 0.27 μmol·L^-1^ ZnSO_4_, 0.001 μmol·L^-1^ CuSO_4_, 0.001 μmol·L^-1^ CoCl_2_, and 0.005 μmol·L^-1^ Na_2_MoO_4_, 0.025 μmol·L^-1^ KI; and pH 6.5.

### Assays of root number and biomass

Wheat root number, including the primary and lateral roots, was evaluated 42 days after sowing (DAS). Ten wheat plants from each treatment group were sampled. The roots were washed with water, and the primary and lateral root numbers were counted. Finally, root samples were dried at 60°C for biomass weight measurements.

### Assays of root O_2_^-^, malondialdehyde content, and superoxide dismutase activity

O_2_^-^ content was determined according to the method described by Bai et al. (2015). Root samples for each treatment were homogenized in 3 mL of 65 mM phosphate buffer (pH 7.8) and the homogenate was centrifuged at 10,000 × g for 10 min. The supernatant (2 mL) was added to 50 mM phosphate buffer (pH 7.8) and 0.1 mL of 10 mM hydroxylamine hydrochloride (0.5 mL). After 20 min at 25°C, the mixture was added to 1 mL of 58 mM sulfanilamide and 1 mL of 7 mM α-naphthyl-amine and incubated at 30°C for 30 min. After this period, the absorbance was measured at 530 nm.

Root samples (0.5 g) were homogenized using a mortar and pestle at 4°C in 5 mL of 50 mmol L^−1^ phosphate buffer (pH 7.8). The homogenate was filtered through muslin cloth and centrifuged at 15000×*g* for 20 min at 4°C, and the supernatant was used to assay the enzyme activities of SOD and MDA content according to Yang et al. (2018). One unit of SOD activity was defined as the amount of crude enzyme extract required to inhibit the reduction of NBT by 50%. The extract (1 ml) and 2 ml of 0.6% thiobarbituric acid were boiled for 20 min and cooled to room temperature. The absorbance of MDA was measured at 600, 532, and 450 nm using a spectrophotometer after centrifugation at 3000 g for 15 min. MDA concentrations were calculated by the equation: MDA (μmol g^-1^ FW) = [6.45 × (OD_532_ – OD_600_) – 0.56 × OD_450_] × V/W, where OD_532_, OD_600_, and OD_450_ are the absorbance at 532, 600, and 450 nm, respectively; V is the volume of extraction, W is the fresh mass of sample.

### Assays of root IAA and ABA content

IAA and ABA were extracted, purified, and measured by high-performance liquid chromatography (HPLC) using the method described by Yang et al. (2018). Root samples (0.1 g) from each treatment were ground to a powder in liquid nitrogen, 4 mL acetonitrile was added, and the homogenate was incubated in the dark at 4°C for 12 h. The extract was centrifuged at 10800 rpm for 10 min at 4°C. The residue was extracted twice with the same solvent. The supernatant was combined and concentrated to residue at 37°C by rotatory evaporation, and re-dissolved in 8.0 mL 0.4 mol L^-1^ phosphate buffer and then added 6.0 mL chloroform and to remove pigment. The pH of the aqueous phase was adjusted to pH3 using pure formic acid. The aqueous phase was extracted thrice with ethyl acetate (3.0 mL). The ethyl acetate phase was concentrated by rotary evaporation and redissolved in 1 mL of the mobile phase. Phytohormone extracts were filtered through 0.2-μm hydrophobic membranes and 10-μL aliquots were injected into a Waters Symmetry C18 column (4.6 mm × 150.0 mm; 5 μm; Waters Corp., Milford, MA, USA) using acetonitrile: methanol: 0.6% acetic acid (5:50:45, v:v:v) as the mobile phase. The flow rate was held at 0.6 mL min^-1^ and the peaks were identified with a photodiode array detector (Waters 2998; Waters Corp., Milford, MA, USA) at an absorbance of 254 nm.

### Co‐expression network analysis for construction of modules of transcriptomics genes

Root RNA extraction, cDNA library construction, Illumina sequencing, read mapping, and differential gene expression analyses have been described previously (Yang et al., 2022). Gene co-expression networks were constructed using the WGCNA package in R software. Network construction and module detection were implemented using the blockwiseModule function. The soft thresholding power was 14. The ‘maxBlockSize’ is ‘nGenes’. The networkType parameter is set to ‘unsigned’. The ‘mergeCutHeight’ was 0.25, and ‘minModuleSize’ was 30. The other parameters were kept at their default values. Modules significantly associated with root physiological traits (root weight, O_2_^-^, MDA, and ABA content) were identified using Pearson’s correlation between eigengene expression profiles and physiological traits. The gene significance (GS) of a gene is defined as the absolute value of the correlation between a gene and a certain clinical parameter, and the module membership (MM) of a gene is defined as the correlation between the module eigengene and the gene expression profile. Genes with higher GS and MM were defined as hub genes in the module. Module networks were visualized using Cytoscape (v3.9.1).

### Statistical analysis and processing

Data for root number and biomass, O_2_^-^, MDA, IAA, ABA content, and SOD activity were processed using DPS v. 7.05. Multiple comparisons were performed using a preliminary F-test. The means were tested using the least significant difference test, and the significance was set at a probability level of 0.05. Graphs were plotted using OriginPro 2017 software (OriginLab Inc., USA).

## Results

### Effect of increasing nitrate supply on root biomass and number

**Figure 1:**
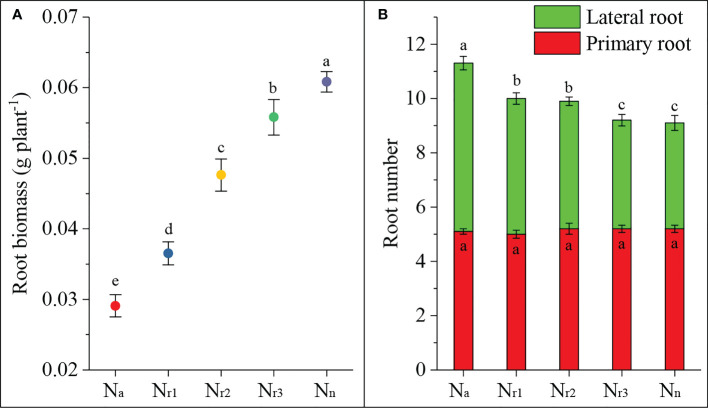
The effects of different NH_4_^+^/NO_3_^-^ ratios on root biomass **(A)** and number **(B)** of wheat root at 42 days after sowing. Segments represent the standard error of the mean (mean ± SE, n = 3). Different letters indicate significant differences among each treatment, *P* < 0.05.

In the present study, we investigated the effects of different NH_4_^+^/NO_3_^-^ ratios on root biomass and primary and lateral root numbers. The results showed that the sole ammonium supply treatment (N_a_) significantly decreased root biomass, but increased nitrate increased biomass (**Figure 1A**). There was no significant difference in the number of primary roots among treatments (**Figure 1B**). But, 42-d of treatment of wheat roots with ammonium significantly increased the lateral root number and total root number. Increasing the nitrate supply decreased the lateral and total root numbers. These results suggest that ammonium can elicit root emergence but inhibit root dry matter accumulation. Increasing the nitrate supply could improve root dry matter accumulation.

### Changes in ROS, MDA content, and antioxidant enzyme activity

**Figure 2:**
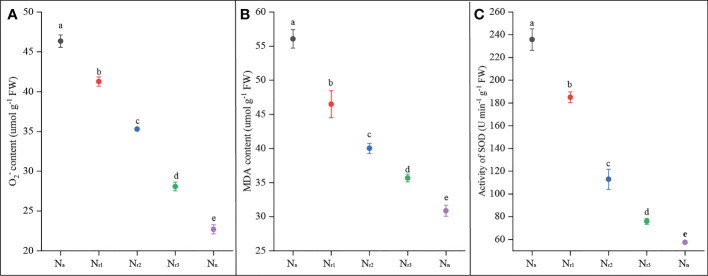
The effects of different NH_4_^+^/NO_3_^-^ ratios on root O_2_^-^ **(A)**, MDA content **(B)**, and SOD**(C)** of wheat root at 42 days after sowing. Segments represent the standard error of the mean (mean ± SE, n = 3). Different letters indicate significant differences among each treatment, *P* < 0.05.

The results showed that root O_2_^-^ and MDA contents gradually and significantly decreased with increasing nitrate supply (**Figures 2A, B**). The N_a_ treatment had the highest O_2_^-^ and MDA content, while the sole nitrate supply treatment (N_n_) yielded the lowest value. Compared with the N_r1_ treatment, root O_2_^-^ and MDA contents under the N_r3_ treatment decreased by 33.12% and 22.21%, respectively. These results suggest that increasing the NO_3_^-^ -N ratio or sole nitrate supply could alleviate ammonium-induced oxidative stress. Roots of ammonium-treated wheat plants had much higher SOD activity, whereas N_n_ treatment had the lowest antioxidant enzyme activities (**Figure 2C**). SOD activity was significantly decreased by increasing nitrate supply. For example, compared to N_r1_ treatment, SOD activity under N_r3_ treatment decreased by 57.72%.

### Transcriptomic genes expression and gene ontology enrichment

**Figure 3:**
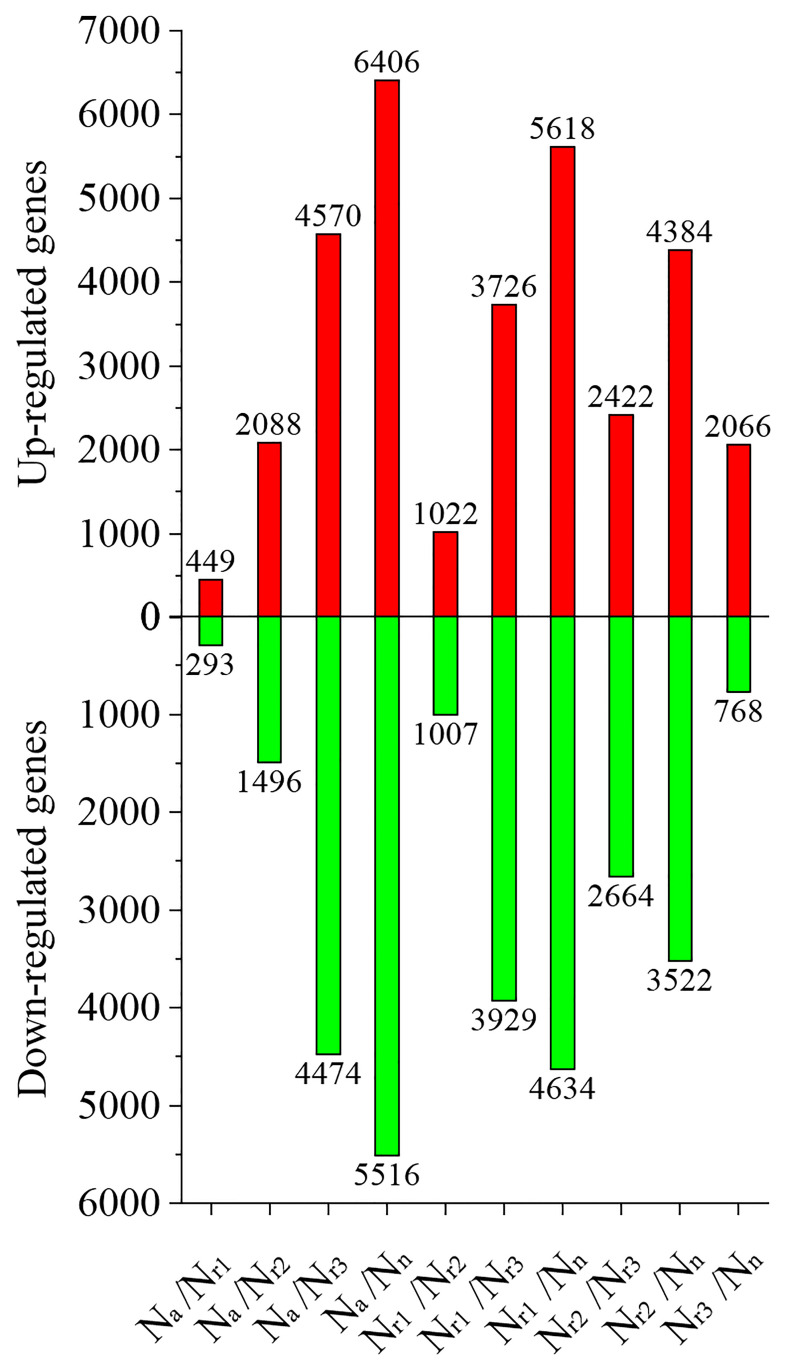
The effects of different NH_4_^+^/NO_3_^-^ ratios on the differentially expressed genes (DEGs) in the wheat root samples.

Next, we analyzed the number of differentially expressed genes (DEGs) under different NH_4_^+^/NO_3_^-^ ratios. For example, 449 upregulated and 293 downregulated genes were identified in the N_a_/N_r1_ group, whereas 4570 upregulated and 4474 downregulated genes were identified in the N_a_/N_r3_ group (**Figure 3**, **Table S1**). A total of 2066 upregulated and 768 downregulated genes were identified in the N_r3_/N_n_ group, whereas 5618 upregulated and 4634 downregulated genes were identified in the N_r1_/N_n_ group.

**Figure 4:**
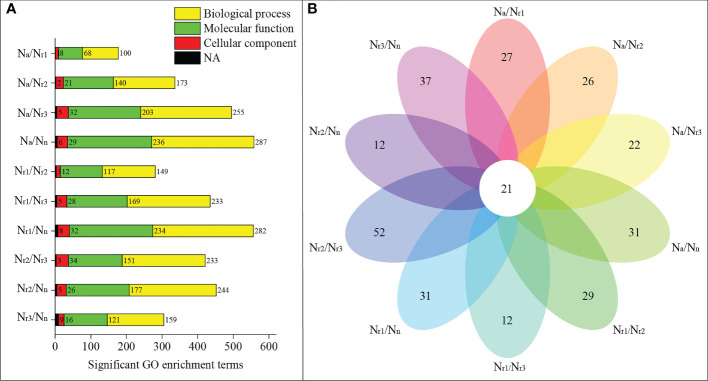
**(A)** Functional enrichment analysis of differentially expressed genes (DEGs) in the wheat root samples of different NH_4_^+^/NO_3_^-^ ratio treatments. **(B)** Venn diagrams represent the number of shared and unique GO terms in each comparisons.

GO enrichment analysis revealed that 558 significant GO terms were identified in the N_a_/N_n_ transcriptome comparison group, which was the highest group (**Figure 4A**, **Table S2**). Only 177 GO terms were identified for the N_a_/N_r1_ group. 31 significant GO terms were unique to N_a_/N_n_, including 16 GO terms enriched in molecular functions, such as glutamate-cysteine ligase activity, and 15 enriched in biological processes, such as the glutathione biosynthetic process (**Table S3**). Additionally, 21 GO terms were shared among all groups, including plant-type cell wall (GO:0009505), peroxidase activity (GO:0004601), nitrate transport (GO:0015706), and phenylpropanoid biosynthetic process (GO:0009699) (**Figure 4B**; **Table S3**).

### Weighted co-expression network analysis

**Figure 5:**
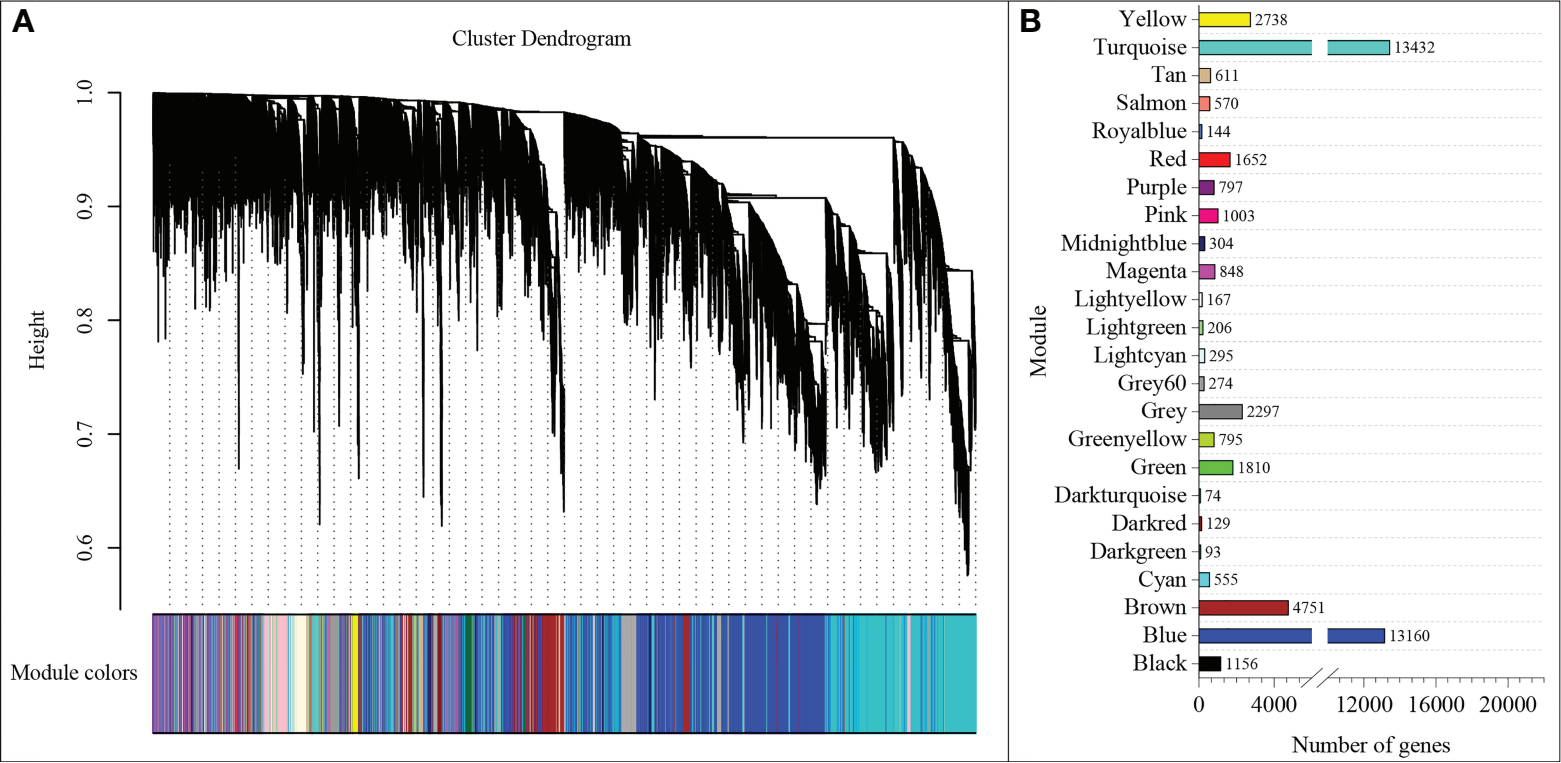
WGCNA of genes **(A)** and modules **(B)** were identified under different NH_4_^+^/NO_3_^-^ ratios treatments.

To understand the regulatory network of increasing nitrate under ammonium stress, we performed WGCNA using the expression data of 85570 genes from the transcriptome data (**Table S4**). The top 75% of the most varying genes selected by a robust covariation estimator relative to median absolute deviation were used as inputs to construct the weighted network. 47861 genes were considered for downstream analysis (**Table S5**). Results showed that a total of 23 distinct modules were identified for the five NH_4_^+^/NO_3_^-^ ratio treatments (**Figure 5**). A total of 23 genes were identified and subsequently defined as hub genes based on the principle of the highest KME (**Tables 1** and **S6**). For example, *TraesCS7B02G271400* was coded as a 40S ribosomal protein in the brown module. *TraesCS6A02G178000* is a plastidic ATP/ADP transporter in the blue module. *TraesCS2B02G056300* was annotated as a WRKY62 transcription factor in the turquoise module. *TraesCS7A02G549000* was annotated as an NAC domain-containing protein 22 in the yellow module.

**Table 1:** Description of hub genes in the modules.

Module	Gene_id	Description
Black	*TraesCS1A02G370100*	Alcohol dehydrogenase ADH3D
Blue	*TraesCS6A02G178000*	Plastidic ATP/ADP-transporter
Brown	*TraesCS7B02G271400*	40S ribosomal protein S24-2
Cyan	*TraesCS1B02G100100*	Hydrophobic protein LTI6B
Darkgreen	*TraesCS2B02G205200*	Hypothetical protein PELPK1
Darkred	*TraesCS6D02G148000*	Nicotianamine synthase
Darkturquoise	*TraesCS3A02G519700*	Hypothetical protein F775_07164
Green	*TraesCS2A02G498600*	Amylogenin
Greenyellow	*TraesCSU02G039900*	Paired amphipathic helix protein Sin3
Grey60	*TraesCS3A02G056000*	GID2 protein
Lightcyan	*TraesCS1B02G432700*	Chloroplast light-harvesting chlorophyll a/b binding protein
Lightgreen	*TraesCS3A02G440100*	Glucuronosyltransferase
Lightyellow	*TraesCS2D02G113000*	Universal stress protein YxiE
Magenta	*TraesCS5A02G449500*	Putative chloride channel-like protein CLC-g
Midnightblue	*TraesCS5B02G189500*	Aminopeptidase
Pink	*TraesCS4A02G401300*	Phenylalanine ammonia-lyase
Purple	*TraesCS3A02G193200*	coatomer subunit beta-2
Red	*TraesCS7A02G155900*	60S ribosomal protein L35a-1
Royalblue	*TraesCS1D02G057600*	hypothetical protein F775_28160
Salmon	*TraesCS2D02G029900*	Cytochrome P450 99A2
Tan	*TraesCS2A02G593300*	NDR1/HIN1-like protein 6
Turquoise	*TraesCS2B02G056300*	Transcription factor *WRKY62*
Yellow	*TraesCS7A02G549000*	NAC domain-containing protein 22

**Figure 6:**
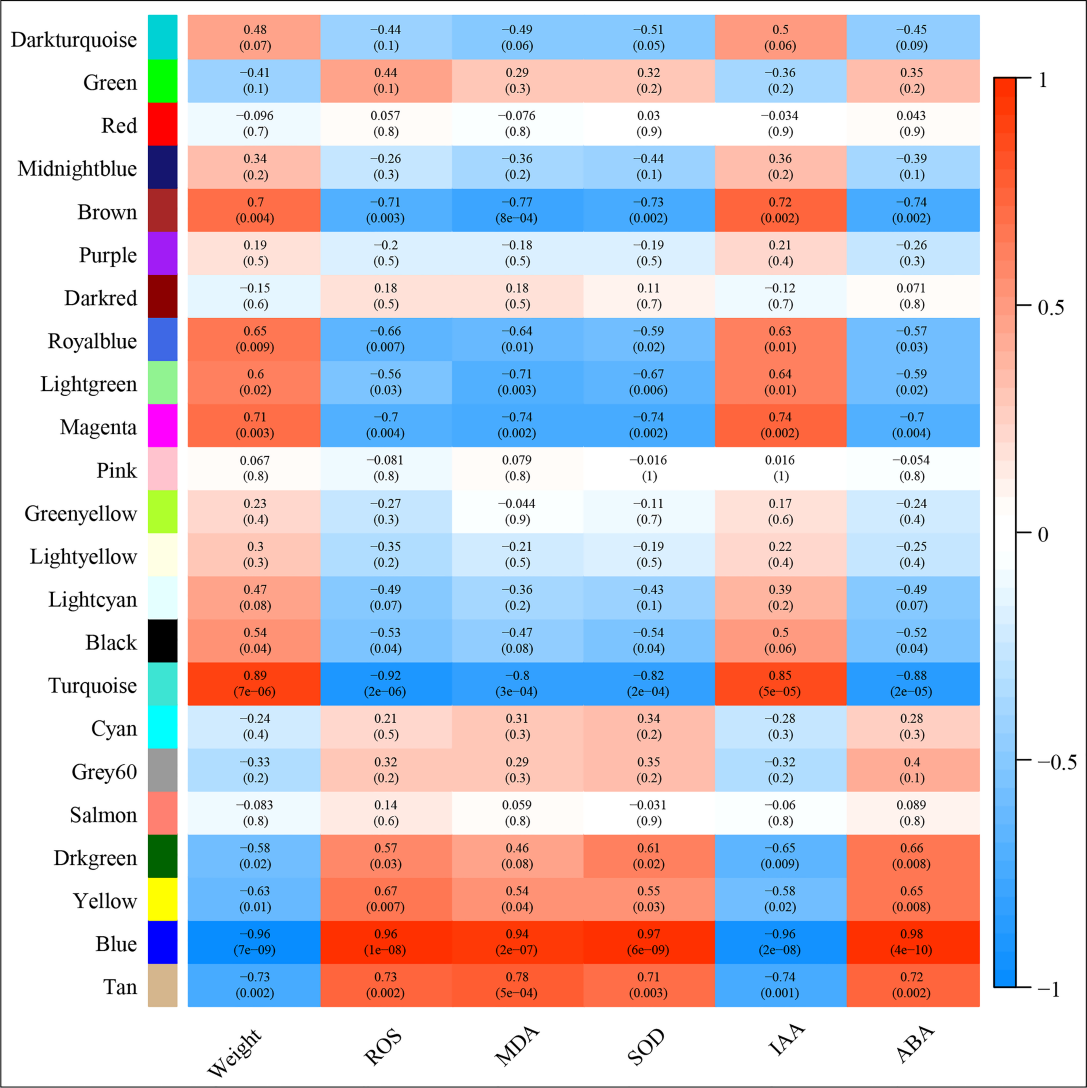
Module‐trait associations. Each row corresponds to a module eigengene, column to a trait. Each frame contains the corresponding correlation and p‐value. The table is color‐coded by correlation according to the color legend.

**Figure 7:**
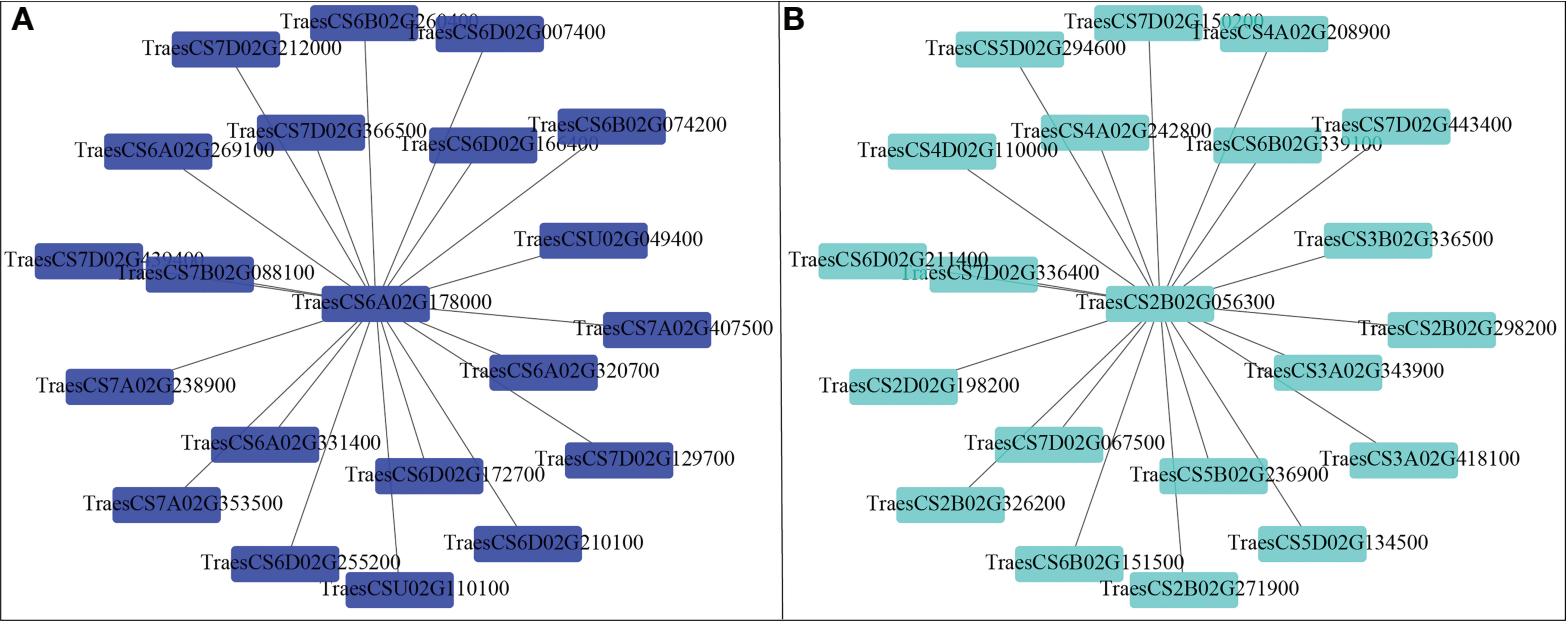
Co-expression network analysis of the hub gene in the blue **(A)** and turquoise **(B)** module.

**Figure 8:**
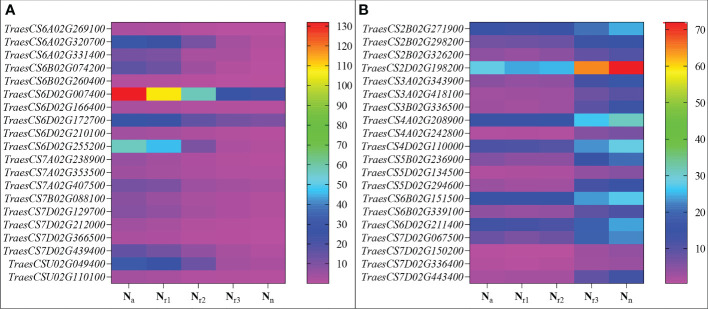
Heatmaps represent the candidate genes expression abundance in the blue **(A)** and turquoise **(B)** module under different NH_4_^+^/NO_3_^-^ ratios treatments. The gene expression was scaled using the fragment per kilobase of transcript per million mapped reads (FPKM) based on a mean value of three biological replicates. Different colors represent the upregulation and downregulation difference of genes among treatments.

Correlation between the modules and root physiological traits, including root biomass, O_2_^-^, MDA, SOD activity, IAA, and ABA content (**Figure 6**). The results showed that the turquoise module was highly related to root biomass. The blue module (with 13160 genes) was highly related to O_2_^-^, MDA, SOD activity, and ABA content. Based on the correlation coefficients, we selected the blue and turquoise modules to perform functional analysis and construct gene networks. According to the TOM values calculated by WGCNA, we constructed a gene co-expression network for the blue and turquoise modules. The top 20 interacting genes were mapped using the weight values (**Figure 7**). The results showed that there were downregulated genes with increasing nitrate supply, which encoded indolin-2-one monooxygenase, Stress responsive gene 1 (SRG1), laccase, ATP/ADP-transporter, Beta3GalT1, DETOXIFICATION, and wall-associated receptor kinase interacting with hub genes in the blue module (**Figure 8A**; **Table 2**). Four upregulated gene transcription factors were identified under nitrate supply conditions: *WRKY24*, *WRKY22*, *JAMYB*, and *MYB30* in the turquoise module (**Figure 8B**; **Table 2**). Genes encoding the CSC1-like protein ERD4, xylan arabinosyl transferase, WD40 protein, NRT1/PTR FAMILY 4.3 were up-regulated by nitrate supply and interacted with hub genes in the turquoise module. In addition, nitrate induced the upregulation of *TraesCS5B02G236900* and *TraesCS2D02G198200* in the turquoise module, which were related to ethylene and gibberellin signal response, respectively.

**Table 2:** Genes associated with hub gene in blue and turquoise modules and their function annotations.

Module	Gene_id	Weight	Description
Blue	*TraesCSU02G110100*	0.3376	Indole-2-monooxygenase
	*TraesCS6A02G320700*	0.3359	SRG1
	*TraesCS6B02G074200*	0.3355	laccase-15
	*TraesCS6D02G166400*	0.3341	ATP/ADP-transporter
	*TraesCS6B02G260400*	0.3302	Beta-1,3-galactosyltransferase Beta3GalT1
	*TraesCS7D02G129700*	0.3292	DETOXIFICATION protein
	*TraesCS6A02G269100*	0.3288	Ubiquinol oxidase 1c
	*TraesCS6D02G210100*	0.3287	Wall-associated receptor kinase 5
	*TraesCS7A02G353500*	0.3274	Aspartate aminotransferase
	*TraesCS6D02G172700*	0.3272	CASP-like protein CASPL
	*TraesCSU02G049400*	0.3268	Metallothionein-like protein type 2
	*TraesCS7A02G407500*	0.3263	Benzyl alcohol O-benzoyltransferase
	*TraesCS6D02G007400*	0.3259	Pathogen-related protein-like
	*TraesCS7D02G439400*	0.3255	Cyanidin 3-O-rutinoside 5-O-glucosyltransferase
	*TraesCS7B02G088100*	0.3253	Sodium/calcium exchanger NCL2
	*TraesCS6A02G331400*	0.3249	Flavonol synthase/flavanone 3-hydroxylase
	*TraesCS7A02G238900*	0.3248	Pyruvate dehydrogenase E1 component subunit alpha-2
	*TraesCS6D02G255200*	0.324	Formate dehydrogenase 2
	*TraesCS7D02G366500*	0.3237	ABC transporter G family member 42
	*TraesCS7D02G212000*	0.3237	UDP-glycosyltransferase 90A2
Turquoise	*TraesCS2B02G271900*	0.3224	CSC1-like protein ERD4
	*TraesCS3A02G343900*	0.3218	WRKY24 transcription factor
	*TraesCS7D02G443400*	0.3213	Carbonic anhydrase
	*TraesCS6B02G339100*	0.3213	Xylan arabinosyl transferase
	*TraesCS7D02G150200*	0.3213	Receptor-like protein kinase
	*TraesCS3A02G418100*	0.3208	JAMYB transcription factor
	*TraesCS4D02G110000*	0.3207	ROH1 protein
	*TraesCS2B02G326200*	0.3204	NRT1/ PTR FAMILY 4.3
	*TraesCS5B02G236900*	0.3202	Ethylene-responsive transcription factor ERF109
	*TraesCS3B02G336500*	0.3200	Uncharacterized gene
	*TraesCS2B02G298200*	0.3197	Uncharacterized gene
	*TraesCS4A02G208900*	0.3195	ROH1 protein
	*TraesCS6D02G211400*	0.3193	MYB30 transcription factor
	*TraesCS7D02G336400*	0.3192	WRKY22 transcription factor
	*TraesCS7D02G067500*	0.3190	Uncharacterized gene
	*TraesCS2D02G198200*	0.3180	Chitin-inducible gibberellin-responsive protein 2 CIGR2
	*TraesCS6B02G151500*	0.3179	Uncharacterized gene
	*TraesCS4A02G242800*	0.3178	WD40 protein
	*TraesCS5D02G294600*	0.3176	L-ascorbate oxidase
	*TraesCS5D02G134500*	0.3175	Cationic amino acid transporter 6

## Discussion

### The physiological effects of increasing nitrate supply on alleviation of ammonium toxicity

Nitrate and ammonium are the main inorganic forms of nitrogen absorbed by plants, but ammonium nutrition often represents an important growth-limiting stressor (Sarasketa et al., 2016). In a previous study, we found that ammonium supply resulted in lower wheat leaf photochemical efficiency and root uptake capacity, thereby reducing plant biomass and leading to stunted growth (Yang et al., 2022). Investigating agronomy management practices and underlining their regulation and signaling processes may contribute to alleviating ammonium inhibition. In the present study, we found that increasing nitrate supply and reducing the NH_4_^+^/NO_3_^-^ ratio inhibited lateral root number, but increased root biomass (**Figure 1**). On the one hand, this may be due to the lower O_2_^-^ content under sole nitrate supply and the low NH_4_^+^/NO_3_^-^ ratio condition (**Figure 2A**). Reactive oxygen species (ROS) are toxic molecules that can cause protein, membrane, and DNA damage. Malondialdehyde (MDA) is a widely used marker of oxidative lipid injury (Davey et al., 2005). Our study showed that the lower NH_4_^+^/NO_3_^-^ ratio treatment significantly decreased MDA content in wheat roots (**Figure 2B**). Furthermore, in the current study, ammonium treatment (N_a_) induced the highest SOD activity, while SOD activity decreased with decreasing NH_4_^+^/NO_3_^-^ ratio (**Figure 2C**). SOD catalyzes the conversion of O_2_^-^ to H_2_O_2_ (Mittler, 2002), which accumulates under ammonium supply and inhibits primary root elongation (Liu et al., 2022). These results indicate that increasing the nitrate supply could alleviate the oxidative stress elicited by ammonium in wheat roots.

**Figure 9:**
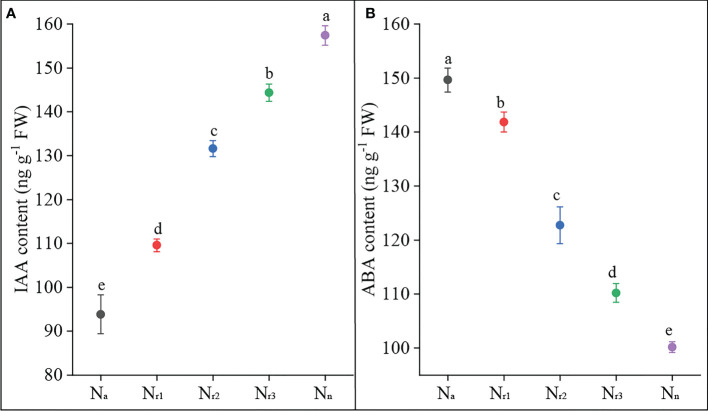
The effects of different NH_4_^+^/NO_3_^-^ ratios on IAA **(A)** and ABA content **(B)** of wheat root at 42 days after sowing. Segments represent the standard error of the mean (mean ± SE, n = 3). Different letters indicate significant differences among each treatment, *P* < 0.05.

On the other hand, nitrate and ammonium supply regulate root system architecture through related hormones, such as auxin and ABA (Patterson et al., 2010; Li et al., 2014; Meier et al., 2020; Liu and von Wirén, 2022). Di et al. (2018) found that high NH_4_^+^ decreased free IAA content in rice roots. Consistent with this result, we found that sole ammonium or high NH_4_^+^/NO_3_^-^ ratio treatment decreased the IAA content of wheat roots (**Figure 9A**). In contrast, increasing the nitrate supply could increase the IAA content. Nevertheless, a previous study stated that high-NH_4_^+^ stress decreased free IAA in roots by increasing IAA inactivation but not by decreasing IAA biosynthesis (Di et al., 2021). Elevated NH_4_^+^ levels can significantly accelerate ABA accumulation in rice tissue ABA accumulation (Sun et al., 2020). ABA plays a direct role in mediating the inhibitory effects of nitrate on lateral root development (Signora et al., 2002). In our study, both sole ammonium and high NH_4_^+^/NO_3_^-^ ratio treatments increased wheat root ABA content, while increasing nitrate supply reduced root ABA content (**Figure 9B**). These results suggest that increasing nitrate supply alleviated ammonium toxicity by regulating root physiological indicators, including SOD activity, ROS, and hormone content.

### The molecular regulation networks of increasing nitrate supply on alleviation of ammonium toxicity

The wheat root is the first organ to be exposed to high NH_4_^+^ concentrations. Previous studies have demonstrated some physiological response mechanisms of ammonium stress from the aspect of transamination ability and oxidative metabolism and identified techniques to alleviate NH_4_^+^ toxicity (Wang et al., 2016; Liu et al., 2021). Molecular adjustment of roots to excess ammonium nutrition may determine the capacity of plants to cope with ammonium stress. In the present study, we combined the transcriptome with physiological responses and used WGCNA to explore functional hub genes and detect the key regulatory factor of nitrate that alleviates NH_4_^+^ toxicity and contributes to root development under ammonium stress. We found that the N_a_/N_n_ comparison group had the most DEGs (**Figure 5**). The results of GO enrichment analysis showed that DEGs were mainly related to plant-type cell wall, peroxidase activity, nitrate transport, and phenylpropanoid biosynthetic processes among all treatments. Further, conjoint analysis of physiological indicators as well as transcriptome data was used to identify root growth-specific gene modules, such as blue and turquoise modules (**Figure 6**). The blue module was strongly related to the ROS and MDA content, SOD activity, and ABA content. The gene encoding the plastidic ATP/ADP-transporter was the hub gene in the blue module (**Table 1**), which was upregulated by the sole ammonium treatment but downregulated with increasing nitrate supply (**Figure 8A**). The main function of plastidic ATP/ADP transporters is to supply ATP to storage plastids (Reiser et al., 2004). Previous studies have suggested that high energy costs of carbohydrate transport and metabolism or futile transmembrane NH_4_^+^ cycling are induced by high ammonium stress (Britto et al., 2001; Ren et al., 2020). In this study, we selected genes that were highly associated with hub genes *via* weight values. For example, *TraesCS6A02G178000* was highly related to the downregulated genes encoding indole-2-monooxygenase, SRG1, DETOXIFICATION 27, CASPL protein, and ABC transporter G family member 42 (**Figure 7A**; **Table 2**). The molecular function of indole-2-monooxygenase shows that it has oxidoreductase activity and can incorporate or reduce molecular oxygen. SRG1 appears to act as a transcriptional repressor and contributes to the engagement of plant defense response (Cui et al., 2018). DETOXIFICATION is also known as a multidrug and toxic compound extrusion protein that is involved in a wide variety of physiological functions throughout plant development. It transports a broad range of substrates, such as organic acids, plant hormones, and secondary metabolites (Takanashi et al., 2014). A previous study found that overexpression of *CASPL* in *Arabidopsis* significantly decreased primary root growth, indicating that CASPL negatively regulates plant growth (Yang et al., 2015). In our study, *TraesCS6D02G172700* encoded the CASPL protein, which was upregulated by ammonium treatment alone and downregulated by increasing nitrate supply (**Figure 8A**). ABC transporters might be a detoxifying mechanism to protect against ammonium stress through the accumulation of N compounds in the root vacuole (Vega-Mas et al., 2017).

In addition, the gene encoding the transcription factor *WRKY62* was the hub gene in the turquoise module and was upregulated by increasing the nitrate supply. Previous studies have reported that *WRKY* transcription factors are among the largest families of transcriptional regulators and play a pivotal role in modulating various signal transduction pathways during biotic and abiotic stresses (Wani et al., 2021). For example, Gao et al. (2018) found that *TaWRKY2* enhances drought tolerance and increases grain yield in wheat. *WRKY62* has been previously reported to be related to disease resistance in rice and Arabidopsis (Kim et al., 2008; Fukushima et al., 2016). In the present study, we found that the turquoise module was highly correlated with root biomass and IAA content (**Figure 6**). As its hub gene, this suggest that *WRKY62* may positively regulate wheat root growth under increasing nitrate supply condition. Previous studies have suggested that hormones and transcription factors cooperate to regulate root growth (Vega et al., 2019; Ortigosa et al., 2021). Ding et al. (2015) reported that *WRKY46* contributes to the feedforward inhibition of osmotic/salt stress-dependent lateral root inhibition *via* the regulation of ABA signaling and auxin homeostasis.

In this study, we constructed correlation networks for *WRKY62* gene (**Figure 7B**). The results showed that *TraesCS2B02G056300* was highly related to the genes that encoded *ERD4*, *ERF109*, *CIGR2*, WD40 proteins, and transcription factors that include *WRKY24*, *WRKY22*, *MYB30*, and *JAMYB*, which were all up-regulated by increasing nitrate supply (**Figure 8B**). Overexpression of the *ERD4* gene could mitigate tobacco plant physiology by enduring stress tolerance (Jha and Mishra, 2021). Cai et al. (2014) reported that *ERF109* integrates JA signalling into auxin pathways to regulate root architecture. *CIGR2* is correlated with the bioactivities of GA and has been reported to play a role in stress responses (Yuan et al., 2016). WD40 interacts with MYB and bHLH to form a ternary regulatory complex (MYB-bHLH-WD40) and could improve tolerance to abiotic stress by regulating root growth and development (Ramsay and Glover, 2005; Kong et al., 2015; Tan et al., 2021). *WRKY24* and *WRKY22* have been identified in the response to salt stress in rice at the seedling stage (Zhou et al., 2016). *JAMYB* overexpression in transgenic Arabidopsis improves tolerance to high-salinity stress during root elongation (Yokotani et al., 2013). *MYB30* is downstream of ROS signalling and regulates root development (Mabuchi et al., 2018). Sakaoka et al. (2018) showed that *MYB30*-regulated root cell elongation is mediated by ROS production under ABA signaling. These studies suggest that increasing the nitrate supply could improve root growth and alleviate ammonium toxicity through molecular regulation networks, including ROS, hormonal crosstalk, and transcription factors.

## Conclusion

In the present study, wheat seedling roots responded to ammonium stress by increasing ROS, MDA, and ABA contents and decreasing root biomass. WGCNA revealed two-hub genes, *TraesCS6A02G178000* and *TraesCS2B02G056300* encode the plastidic ATP/ADP-transporter and transcription factor *WRKY62*, which is related to root growth and ammonium stress, respectively. Increasing nitrate supply alleviated the physiological effects of ammonium toxicity such as a decrease in ROS and ABA content and an increase in IAA content. Further, an increase in nitrate supply down-regulated genes that encoded the CASPL protein and the ABC transporter, and up-regulated genes that encoded *ERD4*, *ERF109*, *CIGR2*, WD40 proteins, and transcription factors including *WRKY24*, *WRKY22*, *MYB30*, and *JAMYB*, which enhanced ammonium tolerance.

## Data availability statement

The original contributions presented in the study are publicly available. This data can be found here: https://figshare.com/articles/dataset/WGCNA/20409792.

## Author contributions

DY conceived and designed the experiments, analyzed the data, prepared figures and tables, authored and reviewed drafts of the manuscript, and approved the final draft. LL, XZ, JL, and JR performed the experiments. All authors contributed to the article and approved the submitted version.

## Funding

The research report here was supported by the National Natural Science Foundation of China (31801295), the Shandong Province Natural Science Foundation (ZR2017BC106), China Postdoctoral Science Foundation funded Project (2018M632701), and supported by China Postdoctoral Science Special Foundation (2019T120600).

## Conflict of interest

The authors declare that the research was conducted in the absence of any commercial or financial relationships that could be construed as a potential conflict of interest.

## Publisher’s note

All claims expressed in this article are solely those of the authors and do not necessarily represent those of their affiliated organizations, or those of the publisher, the editors and the reviewers. Any product that may be evaluated in this article, or claim that may be made by its manufacturer, is not guaranteed or endorsed by the publisher.
