# Supplementary material for: Integrated physiological and weighted gene co-expression network analysis reveals the hub genes engaged in nitrate-regulated alleviation of ammonium toxicity at the seedling stage in wheat (Triticum aestivum L.)
Source: Front Plant Sci. 2022 Nov 17;13:1012966. doi: 10.3389/fpls.2022.1012966 (PMC9713819; doi:10.3389/fpls.2022.1012966)
Supplement: Supplementary file 7 [file Image_1.pdf]

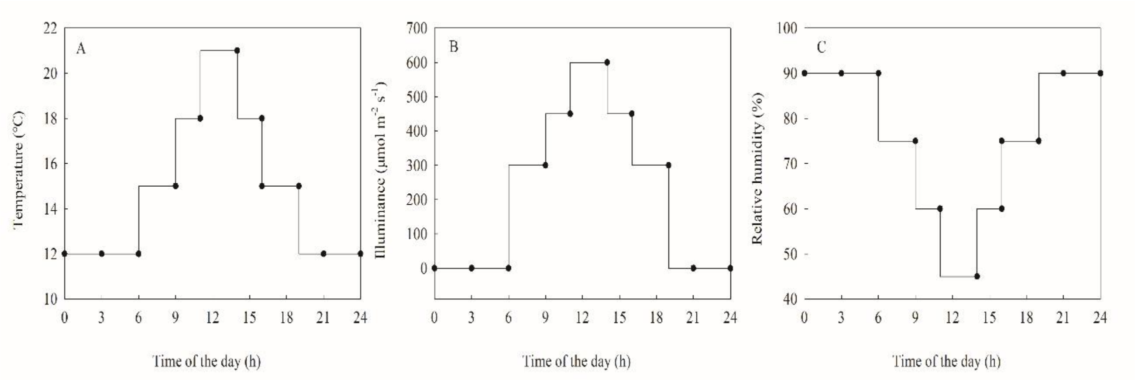

Fig.S1 Daily changes of temperature (A), illumination intensity (B), and relative humidity (C) in the plant growth chamber.
